# Building resiliency in conifer forests: Interior spruce crosses among weevil resistant and susceptible parents produce hybrids appropriate for multi-trait selection

**DOI:** 10.1371/journal.pone.0263488

**Published:** 2022-12-02

**Authors:** Jaroslav Klápště, Barry Jaquish, Ilga Porth

**Affiliations:** 1 Scion (New Zealand Forest Research Institute Ltd.), Rotorua, New Zealand; 2 BC Ministry of Forests, Lands, Natural Resource Operations and Rural Development, Vernon, B.C., Canada; 3 Department of Wood and Forest Sciences, Université Laval, Québec City, Québec, Canada; 4 Institute for System and Integrated Biology (IBIS), Université Laval, Québec City, Québec, Canada; 5 Centre for Forest Research, Université Laval, Québec City, Québec, Canada; Technical University in Zvolen, SLOVAKIA

## Abstract

Tree planting programs now need to consider climate change increasingly, therefore, the resistance to pests plays an essential role in enabling tree adaptation to new ranges through tree population movement. The weevil *Pissodes strobi* (Peck) is a major pest of spruces and substantially reduces lumber quality. We revisited a large Interior spruce provenance/progeny trial (2,964 genotypes, 42 families) of varying susceptibility, established in British Columbia. We employed multivariate mixed linear models to estimate covariances between, and genetic control of, juvenile height growth and resistance traits. We performed linear regressions and ordinal logistic regressions to test for impact of parental origin on growth and susceptibility to the pest, respectively. A significant environmental component affected the correlations between resistance and height, with outcomes dependent on families. Parents sourced from above 950 m a.s.l. elevation negatively influenced host resistance to attacks, probably due to higher *P. engelmannii* proportion. For the genetic contribution of parents sourced from above 1,200 m a.s.l., however, we found less attack severity, probably due to a marked mismatch in phenologies. This clearly highlights that interspecific hybrid status might be a good predictor for weevil attacks and delineates the boundaries of successful spruce population movement. Families resulting from crossing susceptible parents generally showed fast-growing trees were the most affected by weevil attacks. Such results indicate that interspecific ‘hybrids’ with a higher *P. glauca* ancestry might be genetically better equipped with an optimized resource allocation between defence and growth and might provide the solution for concurrent improvement in resistance against weevil attacks, whilst maintaining tree productivity.

## Introduction

To keep pace with the current speed of climate change in terms of sustainable forest productivity, effective management of forest genetic resources must be developed. The assisted gene flow approach implementing transfer functions [1] shifts genetic material from different provenances towards their future optimal growth conditions to maintain the current level of productivity. However, with change in average annual temperature and precipitation, there is also increased frequency of biotic and other abiotic stresses that will require establishing higher tolerance not only to drought, but also to an increased risk of frost damage due to rapid dehardening under a warming climate, and attacks from various pathogens. For Canada, boreal conifer forests are especially at risk for a reduction in timber volume, and by 2100, the unaffected wood volumes are projected to be lower than those currently harvested [2]. Therefore, the implementation of resiliency measures to cope with these disturbances is paramount for a sustainable forestry. Aside from crucial intervention measures for forest insect pests and pathogens, tree breeding for resistance against these forest nuisances is urgent to limit their burden on forest ecosystems.

To achieve tolerance to various stressors, Janes and Hamilton [3] emphasized the importance of interspecific hybrid zones to select individuals with a favourable combination of features from both parental ancestries. Hybridization enhances broader genetic variation and increases the potential for future adaptation which might be important especially under climate change [4]. Additionally, interspecific hybrids are usually more adapted under a broader scale of environmental conditions, while the success of hybridization depends on the phylogenetic distance of the parental species [5, 6]. In some cases, however, there are obvious reproductive barriers due to large phylogenetic distance, causing structural or physiological incompatibility of pollen tubes to establish viable embryos [7]. Therefore, species hybridization is not a universal solution for the generation of resilient genotypes for future environments, and its appropriateness needs to be evaluated on a case-by-case basis.

Spruces are the most important reforestation species in Canada, with hundreds of millions of seedlings planted each year. Interior spruce, a natural hybrid, has a long history of introgression between white spruce (*Picea glauca* (Moench) Voss) and Engelmann spruce (*P. engelmannii* Parry ex Engelm.) as shown by the high levels of recombination events uncovered in hybrid populations [8]. Analyses using genetic markers also revealed asymmetric introgression from white spruce into the local Engelmann spruce species as the initial founder events of the Interior spruce hybrid zone, with eastern British Columbia as the centre of this hybrid zone [8, 9]. Still, the ancestral species maintain their integrity due to environmental selection and limited recent interspecific gene flow (ibidem). In fact, both spruce species occupy distinct ecological niches. While the boreal white spruce has an extremely broad longitudinal range of 111°, the habitat of the mountainous Engelmann spruce is fragmented in western North America covering a much narrower longitudinal range of 23° [8, 10]. Pure species occur below 600 m a.s.l. (*P. glauca*) and above 1,800 m a.s.l. (*P. engelmanii*) according to recent hybrid index assessment using genetic markers [11].

Interior spruce is also one of the economically most important species in western Canada and, due to its hybrid nature, can provide genetic resources needed to cope with climate change [12]. The white pine or spruce shoot weevil (*Pissodes strobi* (Peck); Coleoptera: Curculionidae) is one of the most important biotic threats for Interior spruce [13] and the coastal Sitka spruce (*Picea sitchensis* (Bong.) Carr) species [14] reforestation in western Canada. Similarly, Norway spruce (*P. abies* (L.) H. Karst), which dominates the boreal and subalpine coniferous forests in Europe and has been an important plantation species in eastern North America, is also susceptible to the weevil [15]. The weevil is most damaging to young trees [16]. The white pine weevil’s life cycle starts with adults overwintering in the litter on the ground. From April to May, adults reemerge and start feeding on the terminal shoot. They favour feeding on the bark (phloem tissue) that is close to the dormant terminal buds. Female weevils lay their eggs into the bark of the terminal shoot, and eggs hatch shortly thereafter (7–10 days). The larvae continue feeding on the leader until July when they reach maturity, and adults emerge after 10–15 days and continue feeding on old and new growth [17]. Thus, terminal growth can be severely affected by weevil feeding. Significant losses in productivity due to substantial stem deformations are the anticipated consequence, rendering a spruce plantation meant to provide sawlogs at rotation age worthless. Adding to this, usually it is the tallest and best growing trees that are preferentially infested by the weevil [17]. Adult weevils can persist over several years and the most important natural control of weevil population numbers is through winter mortalities. However, the ongoing shift to milder winters due to climate change might now create the optimal conditions for maintaining higher weevil population numbers and also expand the area of occurrence of the pest, thus increase the frequency of infestation outbreaks. Therefore, resistance against white pine weevil attacks might have to gain higher importance in breeding objectives for spruces.

While conifers have more or less effective defence mechanisms in place [18, 19], the expression of these defences depends on several factors that is the genetic makeup of the host tree and the local environment where host and pest coexist [20]. For example, it was shown for Sitka spruce that synchrony of weevil activity and budburst phenology of the host is determinant for successful oviposition. Faster budburst phenology of the host may contribute to resistance to the white pine weevil [21]. Environmental cues that indirectly influence host preference include overstory shading (shade-grown trees are less preferred [22]) and fertilizer application (fertilized trees due to their increased bark thickness and leader size enhance their attractiveness for weevil oviposition [23, 24]). One of the best studied direct host defences are the bark resin canals related chemical defences [25] that feature oleoresin blends of a great variety of terpenes, generated thanks to an important functional diversity of terpene synthases found in spruces [26]. However, geography and climate may play an important role in the effectiveness of such resin canal defenses, as shown in trees from the northwestern British Columbian zone of introgressions between Sitka and white spruces [27]. Based on the pure species’ biogeography, O’Neill and colleagues concluded that higher *P. glauca* ancestral proportions could mean increased resistance to the weevil in such interspecific hybrids. In general, host tree defences are most effective against local pests, while they weaken under relaxed or absent pest pressure. As the climate warms, these local pest populations may expand their ranges to higher latitudes or elevations, where they may encounter naïve hosts [18], that is hosts that did not coevolve with the pest, since they were never exposed to this threat, and therefore, are less likely to express the appropriate defense response.

The selection of initial plus trees entering the breeding program, and the subsequent selection of parental individuals for advanced generation breeding, has favoured trees with superior growth characteristics. Therefore, screening for resistance of seed sources usually occurs posterior to the detection of a pest or pathogen problem within the breeding program. Therefore, a way to perform mass screening for suitable genotypes is to establish new progeny trials from known seed sources and use artificial infestations to assess genetic control of resistance [28]. These trials can further be used to evaluate how resistance (or tolerance) covaries with growth rate. To do so, the growth rates of tree hosts need to be known before a pest or pathogen occurs to avoid any assessment bias. Long-term breeding programs, such as for the Interior spruce complex *Picea glauca* (Moench) Voss × *P. engelmannii* Parry ex Engelm., exist. It was started in 1973 with a seed collection from 173 ‘plus tree’ Interior spruce individuals growing in natural stands across north central British Columbia (Prince George Selection Unit), where subsequently four progeny test sites involving these open-pollinated (OP) families were established, with subsequent evaluation of the genetic control of juvenile height growth [13, 29]. Furthermore, due to the presence of endemic weevil populations at three test sites, resistance rankings of parents based on retrospective weevil damage assessments were performed over the accumulated sustained damages in each of the 173 OP families [13, 30]. These resistance rankings of parents formed the basis for the selection of parents for the progeny trial at Kalamalka Research station (Vernon, British Columbia) that involved controlled crosses that were generated by mating resistant with resistant, resistant with susceptible, and susceptible with susceptible parents (cross-types; [30]). At 5 years of age (in the fall of 1999), this entire Interior spruce trial was exposed to herbivory through an artificially augmented weevil population occurrence. This established the first large white pine weevil trial for spruces in Canada, and screening for weevil attacks took place from 2000 to 2003 [30]. The outcome of this progeny assessment confirmed for the most part the original parent groupings into resistant or susceptible [13]; certain bark characteristics were confirmed as potential indirect screening tools for resistance in spruce families [30]. However, stable positive genetic correlations between resistance and height growth would be necessary for an effective multi-trait improvement program [13, 31, 32]. First assessments of the relationship between height growth (the directly selected trait) and weevil resistance (the indirectly assessed trait by recorded attacks within one OP family progeny test site with 4,330 trees from 139 families) were conducted from the late 1980ies to the early 1990ies in juvenile Interior spruce [16, 31]. The assessment of attacks took into account the total attacks recorded and three individual years of height assessments during this period. Results indicated overall a strong negative genetic relationship (r = -0.61) between growth and weevil attack. As noticed by the authors, tree heights might also reflect early leader loss and damage for more susceptible families [31]. However, this represents an important confounding factor rendering tree height the result of the level of resistance to pest attack rather than an independent trait. Therefore, the establishment of dedicated progeny trials, where artificial weevil infestations are the tools to screen for weevil resistance and assess whether growth assessed before the attack could be related to subsequent attack severity, becomes crucial [28, 30].

In the present study we revisit the entire Interior spruce weevil resistance screening trial encompassing 2,964 individual genotypes (42 families) of varying susceptibility, established in the interior of British Columbia [30]. The same trial was visited previously by the authors [33–35] to investigate a two-by-two factorial spruce progeny made up of families widely segregating for resistance (resistant-by-susceptible cross-type) and identify individual candidate genes for resistance to the weevil. These studies also pointed at potential pleiotropic relationships between pre-attack growth and pest resistance, thus our interest in revisiting the weevil trial in more detail.

Here, we want to clarify the relationships between resistance to the weevil and, for the first time, pre-attack growth within and across all cross types. We also make direct use of information of parental origin of the selected crosses and investigate whether hybrid status in the studied Interior spruce infestation experiment could be a good predictor for weevil attacks. Our results may have important implications for the future management of breeding programs in Interior spruce, especially when the general environmental gradients under climate change will be considered in addition to the local environmental conditions considered here.

## Material and methods

### Plant material

**Fig 1:**
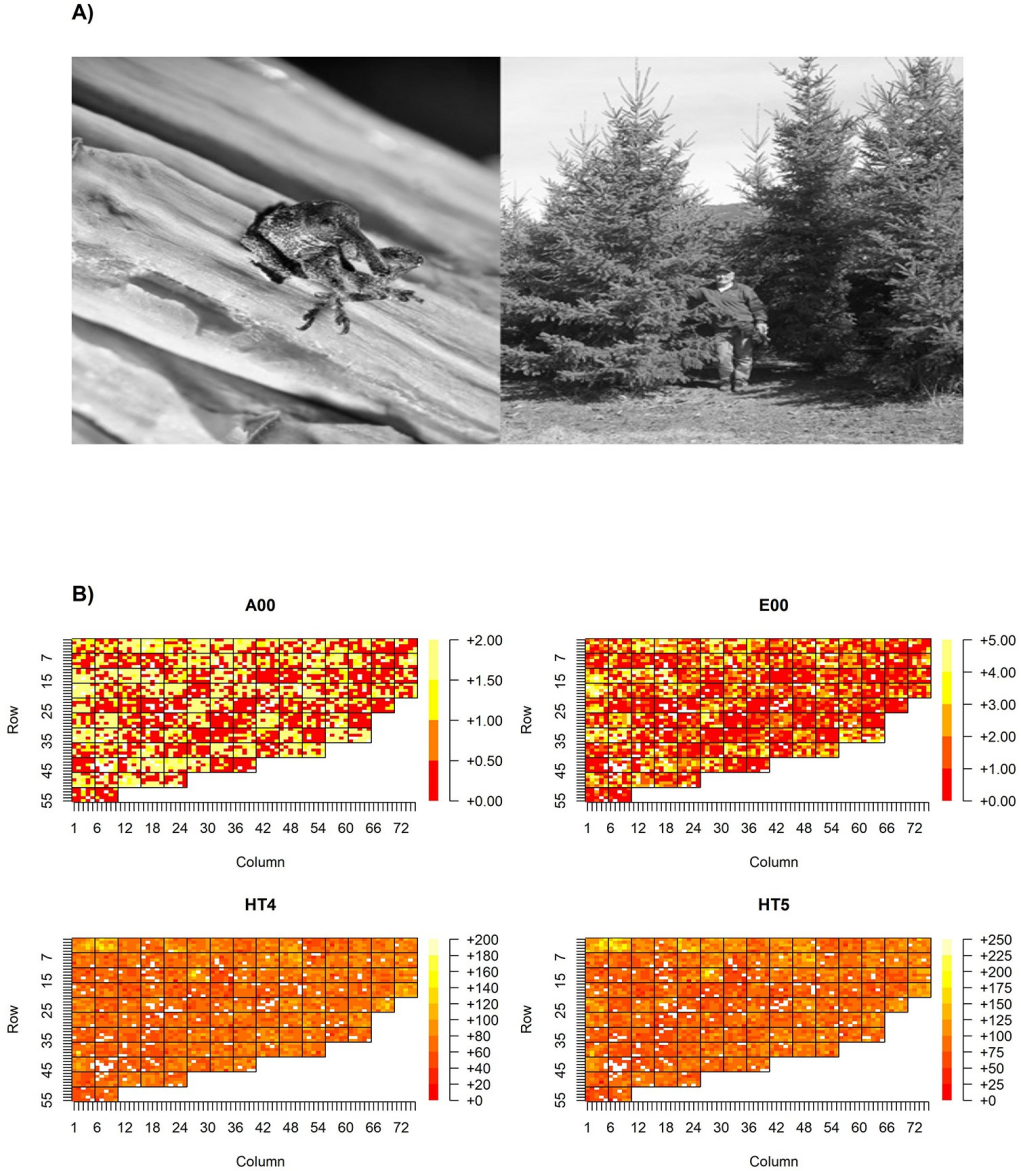
Spruce shoot weevil trial in the interior of British Columbia, Canada. A) Photo to the left: emerging Pissodes strobi individual during the weevil rearing experiment (photo credit: Ward Strong); photo to the right: Kalamalka Research Station based Interior spruce weevil trial after thinning (photo credit: Val Ashley). B) Spatial distribution for classes of attack severity (upper left plot, A00), egg punctures occurrence (upper right plot, E00) in the year 2000, as well as for heights measured in cm at four years (bottom left plot, HT4 in the year 1998) and five years of age (bottom right plot, HT5 in the year 1999), respectively, on an individual tree basis within the experimental plot. Family plots are distinguished in the grid, where individual trees are indexed by row and column numbers. The plot outline of the entire trial on a family basis is provided in [33]. Forty-two controlled crosses were generated by mating resistant with resistant, resistant with susceptible, and susceptible with susceptible parents. The ranking in terms of weevil resistance of the individual Interior spruce parents is known and was done previously [13]. The evaluation of attacks was done following artificial weevil infestation of the spruce resistance trial. Height data from pre-attack years are shown. The colour code for ascending phenotypic values (that is for classes of attack severity, egg punctures occurrence, heights) is provided to the right of each plot outline. No formal statistical analysis on the spatial distribution of the weevil attack severity was attempted for this experiment designed as family-based plots where each plot contained 25 individuals of the same family.

The original plant material for this study was established by crossing Interior spruce (*Picea glauca* (Moench) Voss × *Picea engelmannii* Parry ex Engelm.) parents, previously selected from wild stands and then screened for resistance to the white pine weevil *Pissodes strobi* (Peck) (Fig 1A) attacks through an open-pollinated progeny test [13]. The 13 selected spruce parents (represented as 9 female and 11 male parents in the mating design due to the monoecious nature of spruce) were then clustered into resistant and susceptible groups and three types of controlled crosses (42 crosses in total) were established [30]: 16 families belonging to the RxR cross-type, 20 families belonging to RxS cross-type, and 6 families belonging to SxS cross-type (details are provided in Table 1). In total, 2,964 individuals representing these families were planted in 1995 in a randomized design in multiple plots (each plot contained 25 individuals per family) within three blocks (resulting in a total family size of 75 individuals) at the Kalamalka Research Station in Vernon, BC, Canada (Latitude—50.237222, Longitude—119.274167) (Fig 1B; for the detailed study site layout based on family plots, see [33]). The plant material was assessed for tree heights in 1998 and 1999 at ages 4 yrs and 5 yrs, respectively (HT4, HT5) (Fig 1B); the genetic differences among spruce crosses were assessed by artificial augmentation of the local weevil population in the spruce progeny trial. To achieve this, enough adult weevils were reared from leaders infested with local weevils, and once enough adult weevils had been obtained, three weevils were placed on each of the trees in the trial [30]. Following such artificial augmentation of the local weevil population in October 1999, the annual attack in 2000 (A00) was scored as 0 if there was no attack, as 1 if the attack failed to kill the leader, and as 2 if the attack killed the leader (Fig 1B). This approach clearly revealed the genetic differences by counting the top-kills in each cross, demonstrating the experimental design’s validity. Additionally, the number of egg punctures (i.e., those feeding punctures that have egg covering faecal plugs and thus indicate the extent of successful egg laying along the terminal leader [33]) in 2000 (E00) was recorded and scored as five discrete classes ([34], and Fig 1B). The class variables annual attack and number of egg punctures were transformed to normal scores following [36]. All information on Interior spruce individuals and the raw data is provided in S1 Dataset.

**Table 1:** Mating design representing 42 controlled crosses (families) used in this study.

Mother/Father	1_*R*_	21_*R*_	29_*R*_	87_*R*_	161_*R*_	167_*R*_	1645_*R*_	72_*S*_	79_*S*_	98_*S*_	117_*S*_	128_*S*_	165_*S*_
1_*R*_ (610)	x	RxR	RxR	RxR		RxR	RxR	RxS	RxS		RxS	RxS	RxS
21_*R*_ (732)		x	RxR		RxR	RxR	RxR	RxS	RxS		RxS	RxS	RxS
29_*R*_ (762)			x	RxR	RxR	RxR			RxS				
87_*R*_ (930)				x	RxR	RxR	RxR				RxS		RxS
161_*R*_ (854)					x			RxS	RxS		RxS	RxS	RxS
167_*R*_ (1220)					RxR	x		RxS	RxS				
1645_*R*_ (NA)							x						
72_*S*_ (960)								x					
79_*S*_ (960)								SxS	x		SxS	SxS	
98_*S*_ (1021)										x		SxS	
117_*S*_ (960)											x		
128_*S*_ (1189)												x	
165_*S*_ (1159)								SxS				SxS	x

Details are taken from [30], and the entire trial’s plot outline on a family basis is provided in [33]; since spruce is monoecious, the same tree may serve as the mother or the father in the controlled crosses, as evidenced in the design; R or S indicate the resistance status of the parents with ‘S’ meaning ‘susceptible’ and ‘R’ meaning ‘resistant’ and the resulting cross types RxR, RxS, SxS are also indicated in the table. The elevation in meters above sea level (a.s.l.) is given in brackets next to the maternal parents’ identifier where known.

### Statistical evaluation

The multivariate mixed linear model using MCMC algorithm implemented in the JWAS package [37] was used to obtain the posterior distribution of variance/covariance parameters as follows:

$$\begin{array}{c} Y=X\beta +Zu+e \end{array}$$

where ***Y*** is a matrix of phenotypes (attack, egg punctures, height at ages 4 yrs and 5 yrs), ***β*** is a vector of fixed effects including intercept, disease status in previous year (A99) and replication effect, ***u*** is the vector of random effects including effects of cross, genotype, and plot. Each term in the random effects was modelled by Kronecker product of unstructured variance-covariance matrix and identity matrix. In the case of genotype, the identity matrix was replaced by average numerator relationship matrix [38]. Similarly, residual term e was modelled by Kronecker product of unstructured variance-covariance matrix and identity matrix. The ***X*** and ***Z*** are incidence matrices assigning effects in vectors ***β***, ***u*** to phenotypes of matrix ***Y***. This model was performed for each cross-type separately. The reason for separate analyses by cross-type was based on the fact that separate breeding populations will be needed to also preserve cold-tolerance for instance. Additionally, the joint analysis combining data from all cross-types was performed with the cross-type effect in fixed terms. The model convergence was investigated by the Gelman-Rubin method [39] through merging 5 MCMC runs using 120,000 runs with a burn-in period of 20,000 and thinning of 10 implemented in R package ‘coda’. The posterior estimates of narrow-sense heritability were estimated as follows:

$$\begin{array}{c} h^{2}=\frac{\sigma_{G_{i}}^{2}}{\sigma_{G_{i}}^{2}+\sigma_{E_{i}}^{2}} \end{array}$$

where $\sigma_{G_{i}}^{2}$ is additive genetic variance for *i*^*th*^ trait and $\sigma_{E_{i}}^{2}$ is error variance for *i*^*th*^ trait. Similarly, the posterior estimates of genetic and environmental correlations between traits were estimated as follows:

$$\begin{array}{c} r=\frac{\sigma_{T_{1}T_{2}}}{\sqrt{\sigma_{T_{1}}^{2}\sigma_{T_{2}}^{2}}} \end{array}$$

where $\sigma_{T_{1}T_{2}}$ is the covariance between first and second trait, $\sigma_{T_{1}}^{2}$ and $\sigma_{T_{2}}^{2}$ are the variances for the first and the second trait, respectively. The parameters were extracted from the G1 structure for genetic and the R structure for environmental correlations. Phenotypic correlations were estimated by using the original phenotypic data for continuous variables or normal scores for class variables.

Since Interior spruce is the natural hybrid of white and Engelmann spruce with an elevational hybridization gradient, we tested the statistical significance of the origin of mother/father trees (in terms of elevation) and cross-type on the investigated traits through ordinal logistic regression for class variables (A00; E00) using ‘polr’ function implemented in R package ‘MASS’ as follows:

$$\begin{array}{c} logit\left[P\left(Y\leq j\right)\right]=\alpha_{j}-\sum \beta_{i}X_{i} \end{array}$$

where j is the level of an ordered category (attack severity or egg puncture occurrence) and i is the level of the independent variable (elevation at origin or cross-type) *α*_*j*_ is the intercept or threshold value for *j*^*th*^ level of an ordered category and *β*_*i*_ is the regression coefficient for *i*^*th*^ explanatory variable. Elevation was directly used to order the origin of the parents, while the cross-type was recoded into 0 for SxS, 1 for RxS and 2 for RxR. The proportion of total variance explained by the model was estimated in R package ‘pscl’ as pseudo *R*^2^ by comparing the log-likelihood for the fitted model against the log-likelihood of a null model with no predictors implemented. Similarly, the linear regression was performed for continuous variables (HT4; HT5) using the ‘lm’ function implemented in the R package ‘base’ [40]. The proportion of total variance explained by the model was reported as *R*^2^.

## Results

### Spatial patterns of herbivory and growth

**Fig 2:**
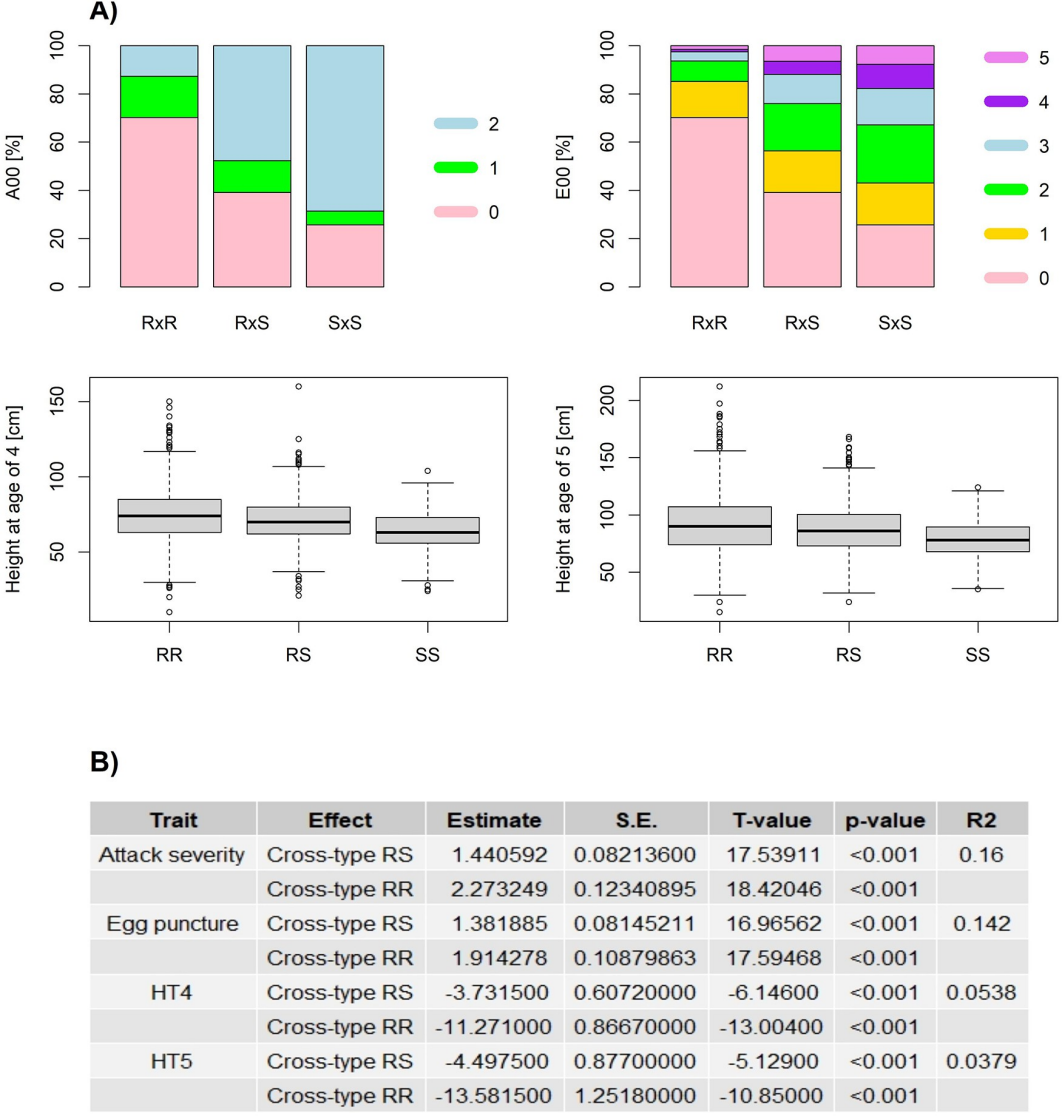
Phenotypic distributions of attack severity, egg puncture occurrences and pre-attack heights within cross-types and their significance. A) Stacked bar charts for weevil attack severity (left, upper plot) and egg puncture occurrence (right, upper plot) in Interior spruce following artificial weevil infestation displayed as the overall percentages for each of the indicated severity levels per cross-type (RxR, RxS, SxS). The colour code for ascending phenotypic values (that is for classes of attack severity, egg punctures occurrence) is provided to the right of both plots. Furthermore, the boxplots diagrams depicting the dispersion of pre-attack heights at 4 years (left, lower plot) and 5 years of age (right, lower plot) are shown for each cross-type of the Interior spruce weevil resistance screening trial. The box in each box plot shows the quartile and the median values of the phenotype dispersion. The whiskers show the range of the phenotypic variation in the individual cross-types. Outliers are depicted as points. B) Statistical significance is shown for cross-type effect on each investigated trait; HT4, HT5: height at 4 years and 5 years of age, respectively; S.E. represents the standard error for the estimates and *R*^2^ the proportion of total variance explained by the model as pseudo *R*^2^ for ‘Attack severity’ and ‘Egg puncture’ and as classical *R*^2^ for ‘HT4’ and ‘HT5’.

The spatial distribution of phenotypes at the study site is displayed in Fig 1B and shows clear patchiness relating to the resistance status of each cross (RxR, RxS, SxS, respectively). The clearest pattern was observed for attack severity where the susceptible crosses exposed excess of attacks while the resistant crosses showed patches of no or low levels of attack (Fig 1B—upper left plot, A00). A similar pattern was observed for the spatial distribution of the egg puncture trait (Fig 1B—upper right plot, E00). This patchiness in the distribution of attack phenotypes was less reflected in heights at both ages. Still, several spruce families emerged as exceptionally high performing (Fig 1B—bottom plots). Given the definitions of resistance of the parental trees (resistant -R- or susceptible -S-) obtained in earlier studies, the weevil attack severity in RxR crosses was the lowest, RxS crosses presented moderate severity, and SxS the highest attack severity (Fig 2A, upper left plot, displayed as the overall percentages for each of the three attack levels). Similarly, the lowest egg puncture occurrence was found in the RxR type while moderate in the RxS type and highest in the SxS type (Fig 2A, upper right plot, displayed as the overall percentages for each of the six egg puncture occurrence classes). Results from ordinal logistic regression showed the statistical significance of cross-type on both attack severity and egg puncture occurrence (Fig 2B).

Additionally, cross-type explained 15% and 12% of the total variance in A00 and E00, respectively (Fig 2B). As expected, the predicted effects of cross-type for each level of attack severity showed an increase in the likelihood of no attack (A00 = 0) while a decrease in the likelihood of severe attack (A00 = 2) with increasing level of cross-type resistance (from SxS (recoded as 0) to RxR (recoded as 2)). On the other hand, moderate levels of attack severity (classified as A00 = 1) did not show any pattern in likelihood related to cross-type (S1 Fig). For egg puncture occurrences (E00), and with increasing level of cross-type resistance, the likelihood for egg puncture level 0 increased while likelihood for all egg puncture levels equal or above 2 decreased. For egg puncture level 1, we did not find any pattern in likelihood related to cross-type (S2 Fig). Heights at ages 4 yrs and 5 yrs followed a similar pattern (Fig 2A, bottom left and right plots), where families in the RxR class showed the highest median values and which gradually decreased toward SxS class. We noticed that the dispersion of values was highest for RxR class, but lowest for SxS class among all three cross types, potentially reflecting the lowest sample size available for SxS class (Fig 2A).

Results from linear regression confirmed the effect of cross-type for height at both years as statistically significant. Yet, the proportion of total variance explained by cross-type reached only from 4% (HT5) to 5% (HT4), which was therefore much lower compared to the total variance explained for the herbivory traits. The increase in mean height was around 5 cm for HT4 and 6 cm for HT5, respectively, with an increasing level of cross-type resistance from SxS to RxR (Fig 2B).

**Fig 3:**
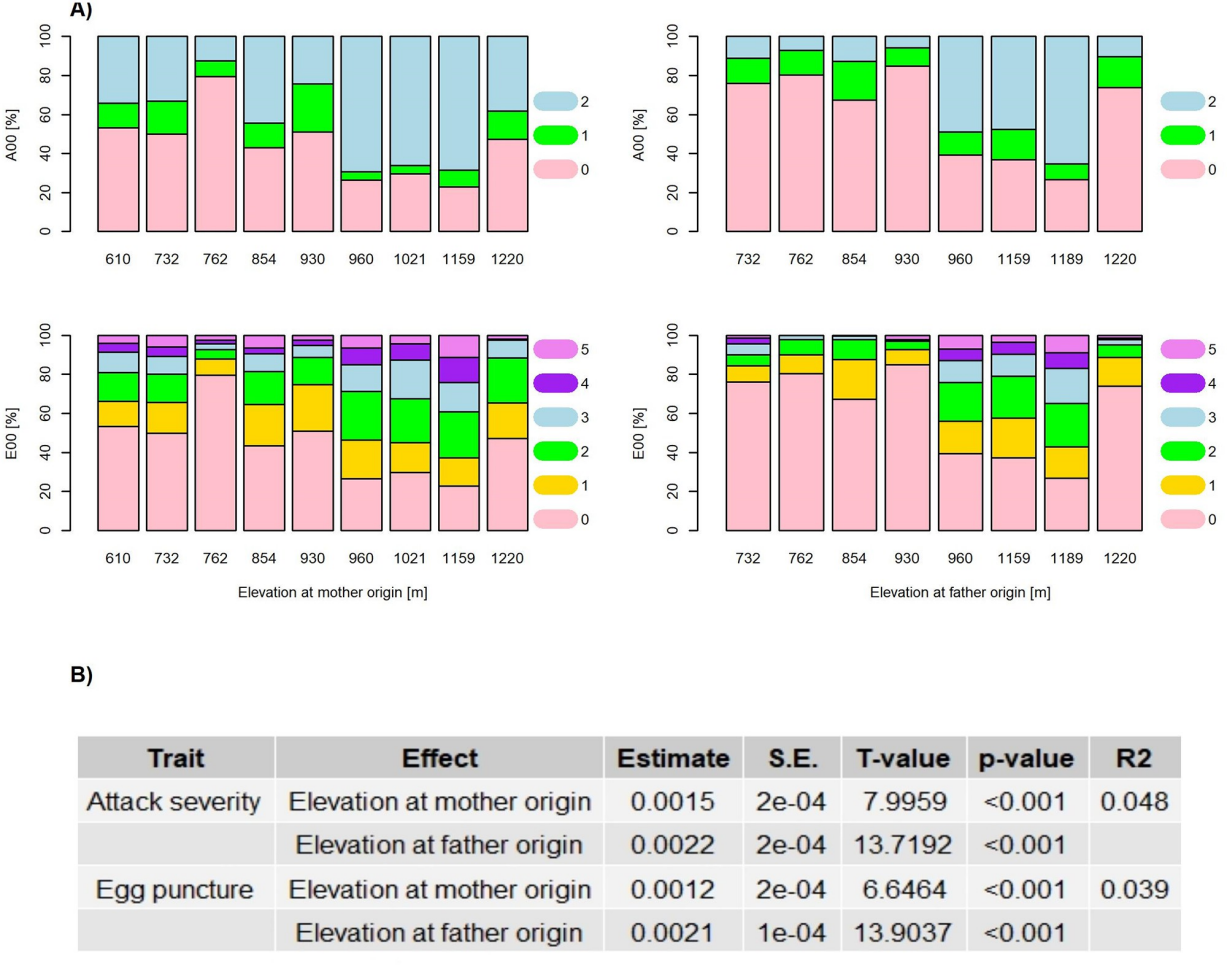
Phenotypic distributions of attack severity and egg puncture occurrences and their significance according to parental origins. A) The distribution of attack severity (A00, upper row) and egg punctures occurrences (E00, lower row) in the Interior spruce progenies (42 families) according to elevation at mother origin (left column) or at father origin (right column) is shown. These 42 controlled crosses were generated by mating resistant with resistant, resistant with susceptible, and susceptible with susceptible parents. The ranking in terms of weevil resistance of the individual Interior spruce parents is known and was done previously [13]. Attack severity increases from 0 to class 2. Egg puncture occurrence increases from 0 to class 5. The evaluation of attack severity and of egg puncture occurrence was done following artificial weevil infestation of the spruce resistance trial. B) Statistical significance of mother/father origin on attack severity and egg puncture occurrence is obtained from ordinal logistic regression. S.E. represents the standard error and *R*^2^ the proportion of total variance explained by the model as pseudo *R*^2^.

Since Interior spruce genetics reflects the natural hybridization between Engelmann and white spruces along an elevation gradient, we investigated whether the distribution of attack severity in the population can be attributed to parental origins. We found that mother trees originating from elevations between 600 m and 950 m had the highest proportion of progeny with no attacks, while mothers originating from elevations between 950 m and 1,200 m had the highest proportion of progeny with severe attacks (Fig 3A, upper row and left column). A similar pattern was observed when the paternal contribution was considered (Fig 3A, upper row and right column). We tested the effect of parental origin on attack severity and found both maternal and paternal effects as statistically significant. Yet, the model explained only 5% of the total variance (Fig 3B). When we plotted the predicted effects of parental origin from ordinal logistic regression, we found that the increase in elevation decreased the likelihood of no attack (A00 = 0) while increased the likelihood of severe attack (A00 = 2). No pattern was observed for moderate attack severity (A00 = 1). These patterns were consistent for both parents (S3 and S4 Figs).

For the distribution of egg puncture occurrences in the population according to parental origin (Fig 3A, lower row), we found a less clear switch in the pattern above 950 m with regards to an impact of parent’s origin. This could be explained by a low number of egg punctures that might have already caused damage to the terminal shoots of the offspring, likely due to a lack of resistance mechanisms passed on from parents coming from high elevation (Fig 3A, lower row). The fact that there was only a small proportion of trees with parental origin at 950 m to 1200 m elevation for which egg punctures did not occur, would exactly reflect such pattern. We also noticed for (especially paternal) parents sourced from 1,220 m elevation a drastic drop in weevil attacks (Fig 3A). Again, these patterns were tested via ordinal logistic regression and found to be statistically significant. The model explained 4% of the total variance (Fig 3B). When the predicted effects of parental origin were plotted for each level of E00, we found that the increase in elevation of parental origin decreased the likelihood of no egg puncture occurrence (E00 = 0), while increased the likelihood at all other levels of E00 (that is E00 = 1 to 5), although the changes were only marginal. Again, such patterns were consistent for both maternal and paternal parent types (S5 and S6 Figs).

### Genetic relationship between herbivory and growth attributes and their inheritance

We assessed heritability, genetic, residual, and phenotypic correlations across and within three distinct cross types (RxR; RxS; SxS). Results are summarized in Table 2. The narrow-sense heritability estimated across the entire population was 0.17 in traits related to herbivory (A00 and E00) and 0.86 and 0.85 related to productivity traits (HT4 and HT5), respectively. When the population was split into cross types, the highest heritabilities were obtained for resistant RxR and susceptible SxS classes ranging from 0.34 to 0.47 in herbivory resistance traits and from 0.51 to 0.86 in growth traits, respectively. The lowest heritability estimates were obtained for the RxS class ranging from 0.13 to 0.14 in herbivory resistance traits and from 0.23 to 0.25 in growth traits, respectively.

**Table 2:** Genetic parameters (correlations; genetic control) were estimated for each cross-type in interior spruce.

Parameter	Trait	All crosses	RxR	RxS	SxS
Genetic corr.	A00-E00	0.53 (0.18)	0.84 (0.12)	0.19 (0.23)	0.46 (0.36)
Genetic corr.	HT4-HT5	0.96 (0.01)	0.91 (0.06)	0.73 (0.14)	0.95 (0.02)
Genetic corr.	A00-HT4	-0.08 (0.45)	-0.74 (0.18)	-0.02 (0.31)	0.16 (0.47)
Genetic corr.	A00-HT5	-0.07 (0.44)	-0.74 (0.18)	-0.03 (0.30)	0.16 (0.48)
Genetic corr.	E00-HT4	0.02 (0.45)	-0.74 (0.18)	0.03 (0.31)	0.29 (0.41)
Genetic corr.	E00-HT5	0.06 (0.44)	-0.73 (0.19)	0.11 (0.30)	0.34 (0.40)
Residual corr.	A00-E00	0.94 (0.02)	0.94 (0.03)	0.94 (0.02)	0.85 (0.21)
Residual corr.	HT4-HT5	0.79 (0.13)	0.95 (0.02)	0.96 (0.05)	0.78 (0.16)
Residual corr.	A00-HT4	0.44 (0.44)	0.75 (0.25)	0.25 (0.09)	0.15 (0.62)
Residual corr.	A00-HT5	0.51 (0.41)	0.75 (0.25)	0.34 (0.08)	0.22 (0.62)
Residual corr.	E00-HT4	0.44 (0.44)	0.76 (0.25)	0.30 (0.09)	0.12 (0.63)
Residual corr.	E00-HT5	0.53 (0.40)	0.76 (0.25)	0.39 (0.07)	0.25 (0.62)
Phenotypic corr.	A00-E00	0.83	0.87	0.80	0.74
Phenotypic corr.	HT4-HT5	0.94	0.95	0.93	0.93
Phenotypic corr.	A00-HT4	0.05	0.12	0.17	0.22
Phenotypic corr.	A00-HT5	0.12	0.16	0.24	0.31
Phenotypic corr.	E00-HT4	0.10	0.18	0.19	0.26
Phenotypic corr.	E00-HT5	0.19	0.24	0.29	0.41
*h* ^2^	A00	0.17 (0.08)	0.47 (0.20)	0.13 (0.05)	0.34 (0.25)
*h* ^2^	E00	0.17 (0.09)	0.45 (0.19)	0.14 (0.05)	0.36 (0.27)
*h* ^2^	HT4	0.86 (0.08)	0.51 (0.17)	0.23 (0.15)	0.86 (0.08)
*h* ^2^	HT5	0.85 (0.08)	0.52 (0.17)	0.25 (0.14)	0.86 (0.08)

A00: attack severity in year 2000; E00: egg punctures in year 2000; HT4: height at 4 years of age; HT5: height at 5 years of age; *h*^2^: narrow-sense heritability; R-resistant; S-susceptible. Numbers in brackets show the estimated standard errors.

Positive genetic correlations were observed between herbivory traits A00-E00 ranging from 0.19 (RxS class) to 0.84 (RxR class) and between growth traits HT4-HT5 ranging from 0.73 (RxS class) to 0.95 (SxS class). The genetic correlations between herbivory resistance traits and growth traits ranged from strongly negative in RxR class (reaching from -0.73 to -0.74) to moderately positive correlations in SxS class (reaching from 0.16 to 0.34). Additionally, the analysis across all cross-types and the RxS class showed a wide range of estimates from low negative to low positive (reaching from -0.08 to 0.11). A similar pattern was observed for the residual correlations with the largest estimates between disease resistance traits ranging from 0.85 to 0.94 and between growth traits ranging from 0.78 to 0.96. The residual correlations between disease resistance and growth traits ranged from strongly positive in the RxR class (reaching from 0.75 to 0.76) to low in the SxS class (ranging from 0.12 to 0.25) and moderate for the entire population analysis (reaching from 0.44 to 0.53) and in the RxS class (reaching from 0.25 to 0.39).

The phenotypic correlations were all positive (Table 2), again, highest between growth (0.94) or between herbivory (0.83) traits and with correlations between A00 and height at both ages highest in SxS crosses (0.22 and 0.31, respectively), followed by RxS (0.17 and 0.24, respectively) and then RxR (0.12 and 0.16, respectively). A similar pattern was observed for E00, but generally, correlations reached higher estimates (Table 2). An important outcome of our re-assessment of the white pine weevil trial was that for the RxR cross-type (Table 2), the sign flipped from highly negative to slightly positive for genetic versus phenotypic correlations between herbivory (A00 and E00) and height traits. Of note is that the respective residual correlations between herbivory (A00 and E00) and height traits in the same cross-type (RxR) were strong and positive, whereas they were otherwise only moderate and positive (RxS and SxS).

## Discussion

### Elevation related to the ancestral origins in interior spruce contributes to the growth and level of resistance against the white pine weevil

The moderate narrow-sense heritability for resistance against the white pine (or spruce shoot) weevil obtained within RxR and SxS cross-type analysis and the relatively high narrow-sense heritability for tree height provides a potential for an effective response to selection for both traits. We stress here that the parental trees deployed in this study were selected solely on the basis of their resistance/susceptibility to white pine weevil attacks and regardless of their origin [13, 30], while our results indicated that susceptible parental trees were predominantly originating from a higher elevation and *vice versa*. The relatively high narrow-sense heritability might result from high genetic diversity captured within the sampled parental trees originating from elevations 610 to 1220 m a.s.l. Since Interior spruce is a natural hybrid between *Picea glauca* and *P. engelmannii* which occupy a transitional elevation zone [9], both species with different growing patterns might contribute to the relatively high narrow-sense heritability. While *P. engelmannii* occupies subalpine environments requiring adaptation for short growing windows and extended winters with deep snow cover, *P. glauca* occupies a boreal environment that competes for light naturally, which selects for relatively fast-growing genotypes. Individuals within the hybrid zone were found to show advantageous features passed on from parental species, such as autumn cold tolerance (*P. engelmannii*) and fast growth (*P. glauca*) [9]. This trend was also observed in this study, where we confirmed that parents of the RxR class that originated from lower elevations, and thus, trees putatively of high *P. glauca* ancestral proportion were tallest (Fig 2). Our hypothesis is based on the observed patterns, while further verification of the population’s actual introgression patterns through genomic markers is needed. We also note that this observation is based solely on pre-attack growth rates. Rossi and Bousquet [41] found that under controlled greenhouse conditions, northern provenances of black spruce showed earlier, and faster bud break compared to southern origins. This result can be seen also as a proxy for the differential local adaptation along an elevation gradient that involves *P. glauca-P. engelmannii* as in the present study, with *P. engelmannii* exhibiting earlier bud burst and budset [9]. Additionally, Wilkinson [42] found a negative genetic correlation between bud break date and early tree height growth. Although De La Torre et al. [9] stated that *P. glauca* does not show growth advantage until it reaches ten years of age, our study found superiority already at 4 years of age in the RxR class, which presumably has the highest *P. glauca* ancestry due to the elevation of origin [11] for the parents involved in RxR crosses (this study). Yet, the differences between cross types explained only ∼4—5% of the total variance (Fig 2B). While the potential of the different genomic introgression patterns among *P. glauca × P. engelmannii* hybrids to explain the differences in the relationship between growth and weevil resistance among individuals has been proposed earlier [35], future research supported by genomic information should be performed to verify the ancestral contribution of the species to individuals in the different cross types and its relationship to the level of resistance/tolerance to white pine weevil attack.

Taking into account the origin of the maternal or paternal parent, the study of attack severity revealed a clear shift from resistant to susceptible genotypes when the parental origin surpassed 950 m a.s.l. (Fig 3). With regards to the co-evolution of the pest with its host, these higher elevations might in fact represent the limit where the short growing window might not allow for completion of the white pine weevil life cycle, and thus, host populations above this elevation are not adapted to this pest. These would thus represent naïve host trees, having never been exposed to the pest. We also found an apparent increase in tree resistance with 1,220 m a.s.l. elevation, thus a pattern which deviated from the general trend. While this result is interesting, it needs to be followed up with a larger sampling of parental trees from elevations around and above 1,200 m a.s.l., but which was not available to us in the current experimental design.

Resistance status was tested in seven crosses, five RxR and two RxS families, which confirmed the resistance segregation pattern expected for a resistant genotype [30]. Since Pinaceae, including spruces, are outcrossing species as evidenced by high levels of heterozygosity in their genomes [43], and the parental genotypes of the F1 generations tested in the trial had directly been sourced from wild stands, the observed resistance pattern above 1,200 m a.s.l. seems valid. We argue that the spring phenology of the hosts might be the determinant factor here. Alfaro et al. [21] found for Sitka spruce that those families resistant to the white pine weevil showed earlier and quicker budburst compared to susceptible families. Generally, individuals at colder sites exhibit greater temperature sensitivity of spring phenology [44]. Therefore, we could assume that families with parental contributions sourced from 1,220 m a.s.l. could show an increased mismatch with the pest’s phenology at the Kalamalka test site, which has a continental climate, and could trigger an accelerated bud flush, since Engelmann spruce and Engelmann spruce-like hybrids require less heat sum [9].

A Québec study performed on 45 Norway spruce genotypes originating from various European provenances, however, found no relationship between budburst phenology, traumatic response intensity and *P. strobi* performance, possibly due to the complete lack of coevolution of this highly sensitive exotic host with the local weevil [45]. The lack of correspondence between the weevil’s biological performance and traumatic resin duct formation in Norway spruce was also suggested in a related study [46]. However, on a broader scale and under a warming climate, the narrowing of phenological mismatches in tree hosts and local insect pests are of concern for the boreal forest [47]. Thus, studies on the state of host-insect phenological synchronies and the forecasted climate-induced phenological changes need to gain in relevance (ibidem). For example, while previous defoliation by herbivores induces earlier budburst in the host as an avoidance strategy [48], warming temperatures again reduce such phenological mismatch due to quicker larval phenology [49]. The implementation of recently available high-throughput assessment methods for tree phenology monitoring that include remote sensing [50] will be crucial to screen trials more holistically and efficiently along trees’ ontogenetic development, thereby offering the possibility to assess phenological changes *in situ*.

### Different resistance mechanisms found between low elevation (RxR) versus low with higher elevation (SxS) crosses

The genetic analysis performed across all cross types discovered weak genetic correlation between tree height and the severity of white pine weevil attack represents a great opportunity for simultaneous genetic improvement for both traits by finding individuals with above-average performance across traits. Such a weak relationship between disease resistance and productivity is rather uncommon. Recent studies showed that tree height is positively correlated with disease resistance such as white pine weevil in Norway spruce [51] or *Leptocybe* gall wasp in *Eucalyptus grandis* [52]. In a differing example, Dungey et al. [53], and later Klápště et al. [54], investigated Swiss needle cast in Douglas-fir introduced in New Zealand. They discovered that while Californian provenances were the most productive on pest-free South Island, their productivity was significantly impacted by Swiss needle cast on North Island, where Oregon provenances were the most prolific due to a higher level of resistance to the pest.

When each cross-type was analyzed separately, opposite patterns in genetic correlations between growth and resistance traits were found. While the RxR class showed strong negative correlations, the genetic correlations for the SxS class were moderately positive. Therefore, fast-growing trees in RxR class were least attacked while fast growing trees in SxS class were mostly attacked. However, the results of the SxS class should be treated with caution due to the small sample size. Additionally, the difference between these two cross types was in the severity of the attack. While the RxR class showed attack severity at level one which indicates that the tree was attacked but that the terminal growth was unaffected, the SxS class resulted in a more severe response (level two) with killed terminal shoots. Similarly to the all-cross-type analysis, the RxS class showed only weak genetic correlations between growth and resistance traits. Surprisingly, a different pattern was observed for the residual compared to the genetic correlations, and these residual correlations were strong and positive for RxR and moderate and positive for RxS and SxS. We argue that a common environment was created by the experimental design where each plot represented multiple individuals that belonged to the same family. This might have generated such rather strong residual correlations (especially in the case of the RxR cross-type), explained by a strong covariance between the permanent environmental effects in the considered traits [55]. For this reason, phenotypic correlations cannot be used in this case as a surrogate of genetic correlations. Where genetic correlations should have the opposite sign, this is mainly assumed for attributes involved in life history evaluation [56, 57], and synchronous growth is one of the life-history traits in forest trees [58]. Again, also in these cases, phenotypic correlations cannot substitute genetic correlations [55].

As one of the defense mechanisms against bark invading insects, conifer trees are releasing oleoresin around the wound to flood deposited eggs, preventing them from further development into larvae and from causing irreversible terminal shoot damage. A significantly higher density of cortical resin canals was found in resistant compared to susceptible genotypes in Sitka spruce [59] and in white spruce [25]. Additionally, the study in white spruce [25] found a positive relationship between tree growth rate and resin canals density. Porth et al. [35] also found that, overall, genetic correlations between bark histology and growth were all positive. For the RxR class, we found indeed those individuals that were fast growing the least attacked. Some families may also display higher tolerance to herbivory, where higher egg puncture occurrence does not automatically result in severe damage outcome for the terminal leader. We found such result, when we regrouped all individual families with strictly 40–80% top-kill outcome [30], where, overall, fast growing trees were less severely attacked and for which genetic correlation between A00 and E00 was extremely low (0.16). However, as stated by Porth et al. [35], the ability to tolerate and at the same time produce well-established chemical defenses towards herbivory might be mutually exclusive, such that we would not expect to observe tolerance in the RxR class.

## Conclusions

Natural disturbances affecting forests are cumulative, highly interdependent, and involve biotic stressors, pests, pathogens individually or in association, and environmental factors, drought, fire alike. All of these depend on climate, conditions, which affect the phenology or life cycle of trees, pests, pathogens. Multi-trait selection is needed to create conifer forest resilience. Tree planting programs need to increasingly consider the consequences of a warming climate on organisms’ phenologies. The resistance to pests and pathogens plays an essential role in enabling tree adaptation to new ranges through tree population movement. This corroborates that resistance breeding programs are continuously important for managing insect pests and pathogen-related diseases. For example, stable positive genetic correlations between height growth and resistance to a local pest would be necessary for an effective multi-trait tree improvement program. Our study on the widely outplanted Interior spruce concluded that selection of fast-growing hybrid genotypes might be the solution for concurrent improvement in resistance against *P. strobi* attacks, whilst maintaining tree productivity. We hypothesize that the RxS hybrid crosses might have inherited high growth rates and dense resin canals from *P. glauca* but also some other features involved in resistance (or avoidance) mechanisms from *P. engelmannii* preventing severe attack of fast-growing genotypes. Such a feature might be a faster rate of bud flush. This is the first study that clearly highlights that hybrid status might be a good predictor for weevil attacks and that also delineates the boundaries of successful spruce population movement. These results that RxS hybrids might be genetically better equipped with an optimized resource allocation between defence and growth has important implications considering the future management of Interior spruce breeding programs and subsequent spruce plantation programs, which need to ensure that adaptation can be maintained across the environment of deployment. Further work is needed to employ genetic markers in efficient and early tree selection.

## Supporting information

S1 Fig Graphical interpretation regarding the ordinal logistic regression for cross-type to explain attack severity (A00). (JPEG)

S2 Fig Graphical interpretation regarding the ordinal logistic regression for cross-type to explain egg puncture occurrence (E00). (JPEG)

S3 Fig Graphical interpretation regarding the ordinal logistic regression for origin of mother to explain attack severity (A00). (JPEG)

S4 Fig Graphical interpretation regarding the ordinal logistic regression for origin of father to explain attack severity (A00). (JPEG)

S5 Fig Graphical interpretation regarding the ordinal logistic regression for origin of mother to explain egg puncture occurrence (E00). (JPEG)

S6 Fig Graphical interpretation regarding the ordinal logistic regression for origin of father to explain egg puncture occurrence (E00). (JPEG)

S1 Dataset (CSV)
