# Correction: Building resiliency in conifer forests: Interior spruce crosses among weevil resistant and susceptible parents produce hybrids appropriate for multi-trait selection

**DOI:** 10.1371/journal.pone.0293160

**Published:** 2023-10-16

**Authors:** The PLOS ONE Staff

There are errors in the Funding section. The correct Funding statement is: I.P. acknowledges financial support from Université Laval from her academic advancement (AA) funds and from an NSERC Discovery grant (ECR supplement funds).

The publisher apologizes for the error.
