# Supplementary figures and images for: Building resiliency in conifer forests: Interior spruce crosses among weevil resistant and susceptible parents produce hybrids appropriate for multi-trait selection
Source: PLoS One. 2022 Dec 2;17(12):e0263488. doi: 10.1371/journal.pone.0263488 (PMC9718410; doi:10.1371/journal.pone.0263488)

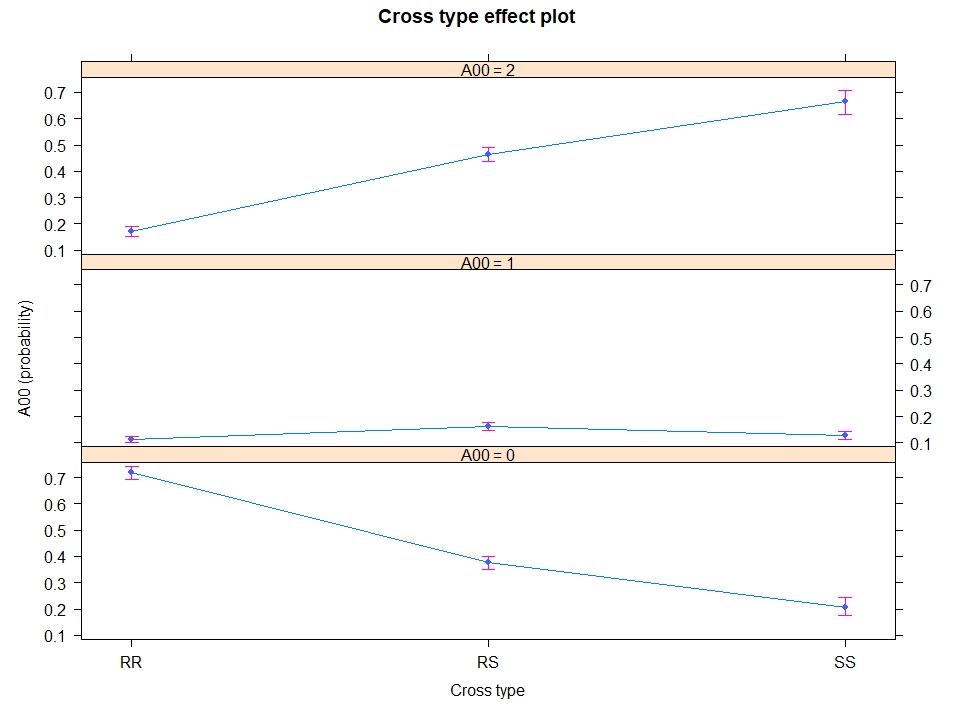

Supplement: S1 Fig — (JPEG) [file pone.0263488.s001.jpeg]

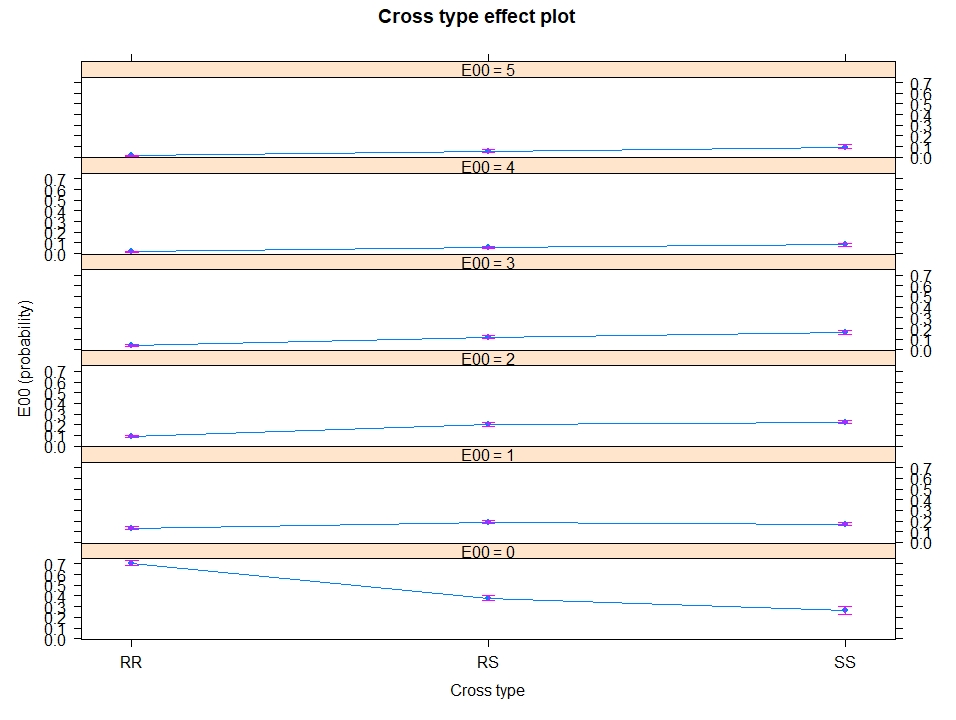

Supplement: S2 Fig — (JPEG) [file pone.0263488.s002.jpeg]

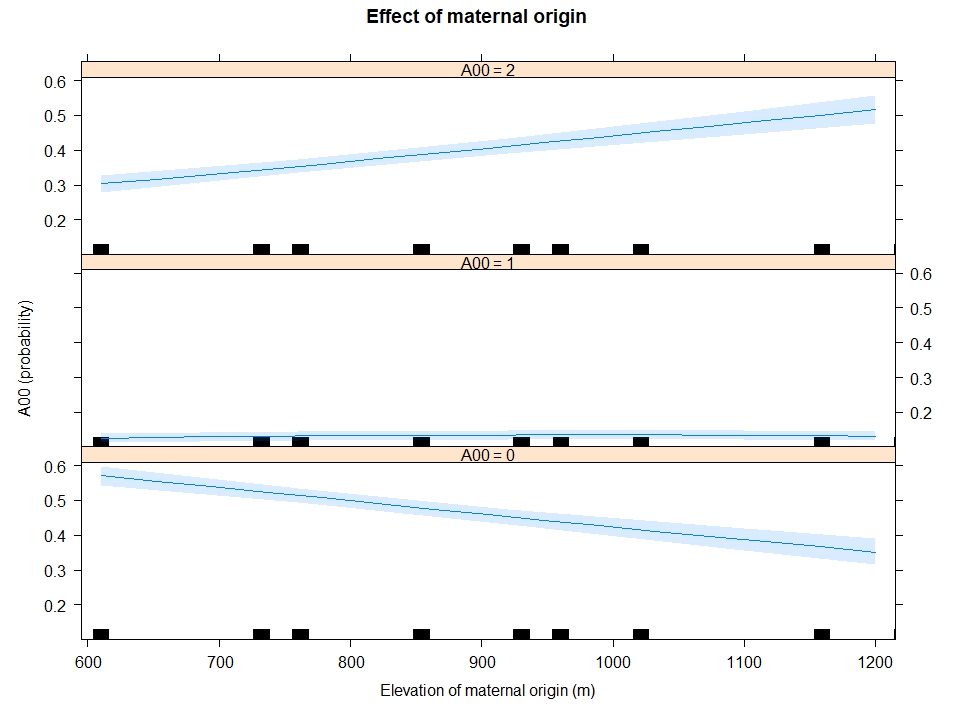

Supplement: S3 Fig — (JPEG) [file pone.0263488.s003.jpeg]

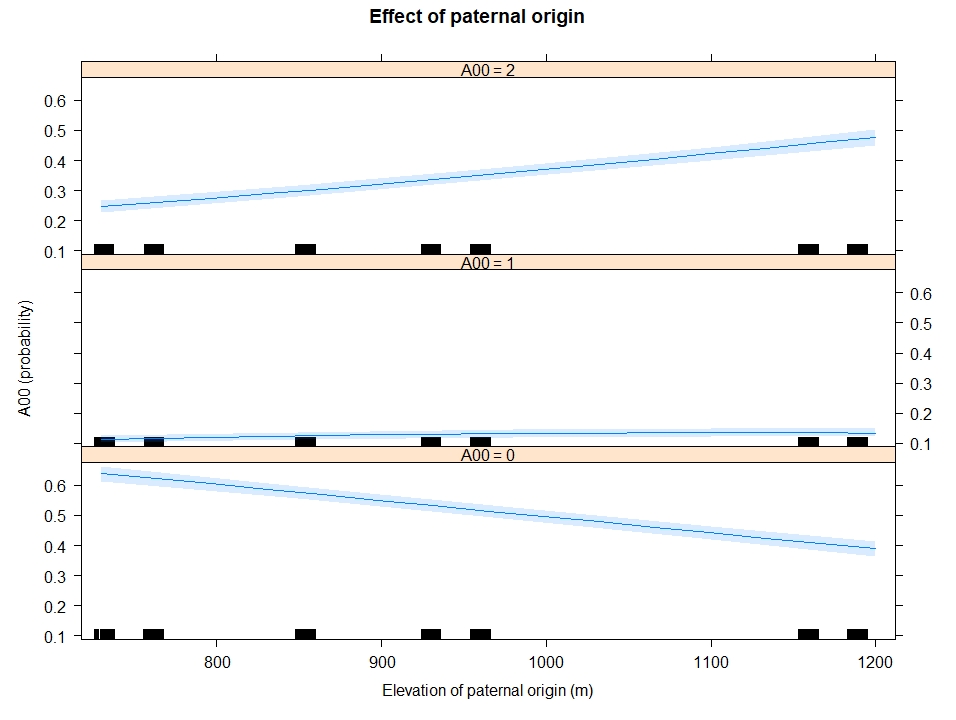

Supplement: S4 Fig — (JPEG) [file pone.0263488.s004.jpeg]

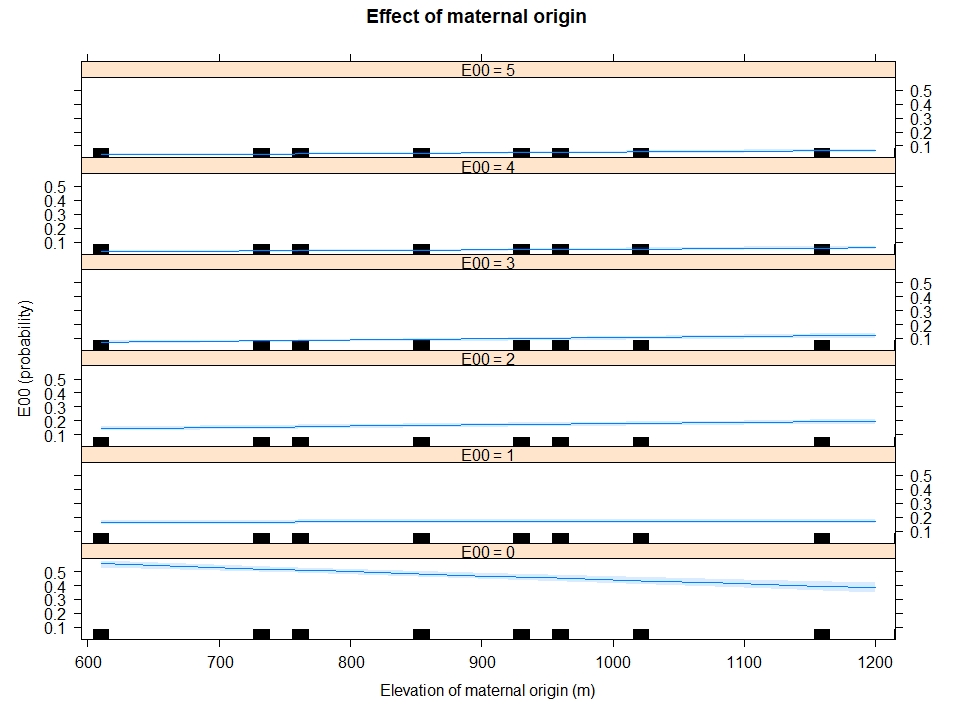

Supplement: S5 Fig — (JPEG) [file pone.0263488.s005.jpeg]

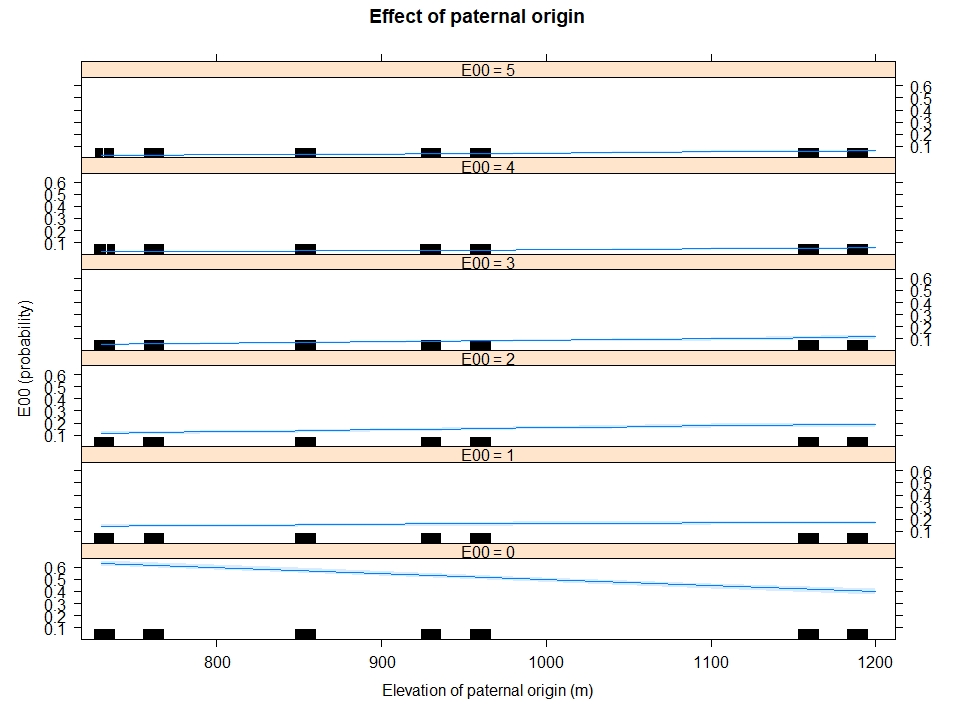

Supplement: S6 Fig — (JPEG) [file pone.0263488.s006.jpeg]
